# Uptake and Retention on Prescribed Psychostimulants for Harm Reduction: A Canadian Cohort Study

**DOI:** 10.1177/29767342261443853

**Published:** 2026-05-09

**Authors:** Paxton Bach, JinCheol Choi, Wing Yin Mok, M-J Milloy, Kora DeBeck, Thomas Kerr, Kanna Hayashi

**Affiliations:** 1 Department of Medicine, University of British Columbia, Vancouver, BC, Canada; 2 British Columbia Centre on Substance Use, Vancouver, BC, Canada; 3 School of Public Policy, Simon Fraser University, Vancouver, BC, Canada; 4 Faculty of Health Sciences, Simon Fraser University, Burnaby, BC, Canada

**Keywords:** methamphetamine, dextroamphetamine, methylphenidate, psychostimulants, amphetamine-related disorders

## Abstract

**Background:**

Methamphetamine use is on the rise in North America and is a growing contributor to the ongoing overdose crisis. Prescription psychostimulants have been available for patients with stimulant use disorder through a provincial harm reduction policy in British Columbia, Canada, since 2020, however, there is little data to inform the uptake of and retention in this intervention in the real-world.

**Methods:**

Data from 3 harmonised prospective cohorts of people who use drugs in Vancouver, Canada, were examined for information on uptake, prescribing patterns, and retention on prescribed psychostimulants provided for the purposes of harm reduction among people who use non-prescribed stimulants. We used a general estimating equations model to examine factors associated with their use.

**Results:**

Among 1005 participants interviewed between 2020 and 2022, 79 (7.9%) received at least one prescription for a psychostimulant as a harm reduction intervention. Daily methamphetamine use and being engaged in some form of addiction treatment were both associated with increased odds of receiving a prescription while later calendar year and age were associated with decreased odds (all *P* < .05). Dextroamphetamine was the most common prescription (70%), followed by methylphenidate (29%). Retention on prescribed psychostimulants was low with only 23% of those receiving a prescription still on the medication at 6-month follow-up.

**Conclusion:**

We observed a low and declining uptake of psychostimulant prescriptions provided for harm reduction purposes during this period, along with poor retention in treatment. These results suggest that further research should investigate factors to improve uptake and engagement of this medication among people who use stimulants, including patient suitability and dosing patterns.

**Highlights**

- The prevalence of stimulant use disorder and its associated harms is rising
- Prescribed psychostimulants are recommended to reduce harm in some settings
- Uptake of prescribing has been low in a cohort of people who use stimulants
- Despite low retention, self-reported satisfaction with prescriptions was positive
- Better data is needed on the effectiveness of this harm reduction practice

## Introduction

The prevalence of illicit stimulant use is rising in North America and is accompanied by a significant rise in both morbidity and mortality.^1,2^ In particular, there is an increasing recognition of the involvement of stimulants (both cocaine and methamphetamine) as significant contributors to the ongoing overdose crisis in Canada and the United States.^3^ This situation is complicated by a paucity of evidence-based treatments for stimulant use disorder, with no currently approved pharmacotherapies and few effective and scalable behavioural interventions.^4-6^ Recent literature and clinical guidelines have, however, highlighted certain medications that may be effective in the management of stimulant use disorders, including prescribed psychostimulants.^7-9^

In the province of British Columbia, Canada, the emergence of the COVID-19 pandemic triggered a host of new public policies designed for the dual purpose of (1) helping to reduce risk of coronavirus infection, and (2) preventing overdose among people who use drugs (PWUD).^10^ These policies included guidance on the use of “prescribed safer supply” (also known as “prescribed alternatives to the toxic drug supply”, hereafter referred to as “safer supply”), which are harm reduction interventions intended to help promote self-isolation as well as decrease risk of overdose by reducing reliance on an increasingly potent and unpredictable unregulated drug supply now dominated by fentanyl and other synthetic opioids.^11^ Among the recommended medications were both dextroamphetamine and methylphenidate, for use in people who have been diagnosed with stimulant use disorders. However, data on their uptake and impacts remains lacking.

The present study set out to examine the prevalence, prescription patterns, self-reported barriers, retention, and patient satisfaction around access to prescribed psychostimulants in a community-recruited prospective cohort of PWUD in Vancouver, Canada, between 2020 and 2022.

## Methods

### Study Setting and Participants

Data were collected from 3 complementary, ongoing open prospective cohorts of community-recruited PWUD in Vancouver, which has been described in detail previously: the Vancouver Injection Drug Users Study (VIDUS); the AIDS Care Cohort to evaluate Exposure to Survival Services (ACCESS); and the At-Risk Youth Study (ARYS).^12-14^ Briefly, they are composed of HIV-negative participants who have used injection drugs within the month prior to enrollment (VIDUS), people living with HIV who have used any unregulated substance (other than cannabis) in the month prior to enrollment (ACCESS), and street involved youth between the ages of 14 to 26 years old at the time of recruitment who have used any unregulated substance (other than cannabis) in the month prior to enrollment (ARYS). The cohorts employ harmonised data collection protocols, using the same questionnaire at the same follow-up frequency, to allow for merging of data. Participants are only eligible to participate in a single cohort at any given time, though may transition between them as their HIV status changes. At the time of enrollment, participants complete an interviewer administered by trained research staff, with follow-up interviews every 6 months thereafter. During the current study period, all interviews were conducted remotely (mostly via phone) due to the COVID-19 pandemic-related public health restrictions.^15^

For the present study, the sample was restricted to participants from all studies who reported any stimulant use (crack cocaine, powder cocaine, and/or crystal methamphetamine) in the past 6 months and completed at least one study visit between July 1, 2020 and November 30, 2022. This sample was selected to capture all participants who report active stimulant use, though not all of these participants might meet diagnostic criteria for a stimulant use disorder (and thus be eligible for such an intervention).

### Measures

The primary outcome for this study was self-reported receipt of a prescribed psychostimulant specifically indicated as a pharmaceutical alternative to the toxic drug supply at any point during the study period (yes vs no). A range of explanatory variables were included based on a known or hypothesised association with the primary outcome, specifically: calendar year of interview, age (per 10 years); gender; self-identified race**/**ethnicity/ancestry; homelessness; residing in the Downtown Eastside of Vancouver (an urban neighbourhood with a high concentration of community resources and substance use); regular employment; involvement in sex work; and a history of psychosis (ever). Drug use patterns included ≥daily non-prescribed opioid use; ≥daily cocaine and/or crack use; ≥daily methamphetamine use; ≥daily injection drug use; HIV status; recent non-fatal overdose; and current enrollment in addiction treatment (a 3-level variable consisting of opioid agonist therapy (OAT) ± opioid safer supply vs any other form of substance use treatment vs none [reference category]). Other indicators of socio-structural exposures included the reported inability to access addiction care (defined as at least one unsuccessful attempt within the past 6 months). All behavioural variables referred to activities within the previous 6 months.

### Statistical Analyses

Bivariate generalised estimating equations (GEE) with a logit link and an exchangeable correlation structure (to account for within-subject correlation) were employed to estimate unadjusted associations between the explanatory variables and reported receipt of a psychostimulant prescription.

In subanalyses, descriptive statistics were used to examine medication(s), doses, and participant satisfaction with prescribed psychostimulants (measured by a 5-point Likert scale). For those who did not receive a prescription, we examined self-reported reasons for why a prescription was never received (selected from a prepopulated list of hypothesised reasons, with the ability to provider “other” responses by narrative text). Among those who received a psychostimulant prescription at one study visit, we also examined proportions of those who continued to receive the prescription at subsequent study follow-ups.

All *p*-values were two-sided, and statistical analyses were conducted in R software version 4.4.1 (R Foundation for Statistical Computing, Vienna, Austria) and SAS software version 9.4 TS Level 1M6 (SAS, Cary, NC, USA). As an exploratory study with limited statistical power, no corrections for multiple comparisons were made; thus, results should be interpreted with caution.

## Results

A total of 1005 participants who reported stimulant use within the past 6 months were included in the study sample including 402 (40%) identifying as women, 556 (55%) of white ethnicity, and 394 (39%) of Indigenous ancestry. The median number of follow-up visits was 3 per participant over the study period. At baseline, the median age of participants was 43.2 years (interquartile range [IQR] 32.2-54.5), 364 (36%) reported daily injection drug use, 139 (14%) reported daily cocaine and/or crack use, and 260 (26%) reported daily methamphetamine use. Further details on the study sample characteristics are shown in Table 1. A total of 50 participants (5.0%) reported receipt of a prescription for psychostimulants as a pharmaceutical alternative at baseline, and 79 participants (7.9%) reported a prescription at any point during the study period.

**Table 1.** Baseline Characteristics and Bivariate Analysis of Factors Associated With Receiving a Prescribed Psychostimulant in the Previous 6 Months (n = 1005).

Characteristic	Psychostim Rx n = 50 (5.0%)	No Psychostim Rx n = 955 (95.0%)	Odds ratio (95% CI)
Age (median, IQR)	34.6 (29.3-45.4)	43.6 (32.6-54.8)	0.96 (0.94-0.98)*
Ethnicity/ancestry			
White	29 (58.0)	527 (55.2)	1.66 (0.99-2.80)
Indigenous/person of colour	19 (38.0)	422 (44.2)	
Gender			
Woman	18 (36.0)	384 (40.2)	0.87 (0.53-1.42)
Man/non-binary/other	32 (64.0)	571 (59.8)	
Homelessness^a^			
Yes	13 (72.0)	189 (19.8)	1.23 (0.71-2.12)
No	36 (26.0)	762 (79.8)	
Reside in Downtown Eastside^a^			
Yes	27 (54.0)	489 (51.2)	1.29 (0.81-2.04)
No	21 (42.0)	459 (48.1)	
Recent employment^a^			
Yes	20 (40.0)	301 (31.5)	1.02 (0.69-1.50)
No	30 (60.0)	652 (68.3)	
Sex work involvement^a^			
Yes	6 (12.0)	123 (12.9)	1.23 (0.69-2.20)
No	43 (86.0)	829 (86.8)	
Daily non-prescribed opioid use^a^			
Yes	31 (62.0)	480 (50.3)	1.32 (0.82-2.13)
No	19 (38.0)	473 (49.5)	
Daily cocaine and/or crack use^a^			
Yes	4 (8.0)	135 (14.1)	0.64 (0.37-1.11)
No	46 (92.0)	813 (85.1)	
Daily methamphetamine use^a^			
Yes	24 (48.0)	236 (24.7)	2.20 (1.40-3.45)*
No	26 (52.0)	715 (74.9)	
Daily injection drug use^a^			
Yes	23 (46.0)	341 (35.7)	1.54 (0.97-2.43)
No	27 (54.0)	608 (63.7)	
HIV			
Positive	9 (18.0)	297 (31.1)	0.74 (0.42-1.29)
Negative	41 (82.0)	658 (68.9)	
History of overdose^a^			
Yes	11 (22.0)	191 (20.0)	1.43 (0.94-2.17)
No	39 (78.0)	756 (79.2)	
Addiction treatment^a^			
OAT ± opioid safer supply	40 (80.0)	518 (54.2)	7.66 (3.42-17.16)*
Other treatment	6 (12.0)	61 (6.4)	9.08 (3.58-23.03)*
No treatment	4 (8.0)	371 (38.9)	REF.
Unable to access treatment^a^			
Yes	5 (10.0)	53 (5.6)	1.56 (0.79-3.06)
No	45 (90.0)	892 (93.4)	
History of psychosis			
Yes	6 (12.0)	40 (4.2)	1.82 (0.72-4.61)
No	43 (86.0)	914 (95.7)	

Abbreviations: IQR, interquartile range; OAT, opioid agonist therapy.

a Denotes activities or situations referring to the past 6 months.

* *P* < .05.

In the bivariate GEE analyses daily methamphetamine use (odds ratio [OR] = 2.20, 95% confidence interval [CI] 1.40-3.45), OAT and/or safer opioid supply prescriptions (OR = 7.66, 95% CI 3.42-17.16), and other addiction treatment (OR = 9.08, 95% CI 3.58-23.03) were positively associated with receiving a prescription. Age (per 10 years older; OR = 0.96, 95% CI 0.94-0.98) and calendar year of interview (OR = 0.73, 95% CI 0.59-0.91) were negatively associated with a prescription (Table 1).

Among those who did receive a prescription for psychostimulants as a pharmaceutical alternative over the study period, the most common medication received was dextroamphetamine (either immediate-release, extended-release, or a combination of both; 70%), followed by methylphenidate (also either immediate-release, extended-release, or a combination of both; 29%). The mean daily dose of dextroamphetamine, as reported by participants, was 46.9 mg, and the mean daily dose of methylphenidate was 51.5 mg. Among participants who did not receive prescriptions, the most commonly reported reasons for this were “not interested” (493; 53%), “did not know about prescribed safer supply guidelines” (338; 37%), and “other” (95; 10%).

At the time of most recent follow-up where a prescription was received, 43% (n = 33) of recipients that responded to this question (n = 77) reported that they were “satisfied” or “very satisfied” with the medication that they had received, while 14% (n = 11) were “neither satisfied nor unsatisfied,” 22% (n = 17) were “unsatisfied” or “very unsatisfied,” and 21% (n = 16) did not respond or did not know. Retention on any prescribed psychostimulant over the duration of the study was low, with only 23% (18/79) who had received a prescription reporting an ongoing prescription at the first 6-month follow-up, 5% (4/79) reporting an ongoing prescription at the second follow-up, and 3% (2/79) reporting an ongoing prescription at third follow-up. The number of participants within this sample accessing a prescription for a psychostimulant per follow-up period declined over the course of this study, dropping from 5% to 3% overall.

## Discussion

This study describes the prevalence of and factors associated with prescribed psychostimulant use for harm reduction purposes among a sample of people who used unregulated stimulants during the overlapping public health crises of overdose and the novel coronavirus pandemic. A relative minority of participants reported having received a prescription at any point in time, with lack of interest and/or awareness of their availability reported as the most common reasons for not having received a script. Notably a lack of contact with prescribers or an inability to obtain a prescription were not highlighted as common reasons for not receiving a prescription, despite these being available survey responses.

In general, this study aligns with other research on the British Columbia prescribed safer supply policy, which has demonstrated a significantly greater uptake in opioid prescribing compared to psychostimulants (91% of total recipients of “safer supply” compared to 18%).^16^ This may reflect a higher perceived need (ie, higher risk of overdose) for those who use opioids, and/or relate to prescribers’ perceptions or experiences of efficacy with prescribed opioids compared to psychostimulants (potentially corroborated by the reported difference in overdose death reduction between the 2 classes, which was significant only for those who were receiving opioid prescriptions).^16^ Alternatively, the fact that 37% of participants reported that they were unaware of prescribed safer supply guidelines is consistent with our previous study and may suggest a need for increased education and engagement efforts.^17^

While the prevalence of prescribed psychostimulants was relatively low in this population, those who did access prescriptions had a moderately high satisfaction with their medications (43% of recipients reported being either “satisfied” or “very satisfied”). Insight into factors that might improve reported satisfaction can be obtained from a recent qualitative study performed in a similar population, which suggested that medication type, dose, and ease of access were all directly related to participants interest in engagement.^18^ It is noteworthy that satisfaction was this high given a recent meta-analysis suggesting that, for these medications, daily doses of 60 mg or more were required to promote abstinence from other stimulants, while in our study, the average doses were significantly lower.^9^ Subtherapeutic doses may also be a contributor to the poor overall retention in treatment from one study follow-up period to the next, though clinical trials have not examined the efficacy of these medications over time and this may equally relate to a lack of durability in the therapeutic effect.

This study adds to a limited body of knowledge around real-world experiences with psychostimulant prescribing in the context of stimulant and other substance use disorders. The literature is currently scant in this area and presents conflicting results. One large Swedish study suggests that the psychostimulant lisdexamfetamine was uniquely associated with a reduced risk of hospitalisation and all-cause mortality amongst a population of individuals with amphetamine use disorders, while others have reported beneficial outcomes of psychostimulant prescriptions on OAT retention.^9,19,20^ Conversely, risk of drug poisoning has also been shown to be associated with receipt of a psychostimulant prescription, demonstrating the complexity of these real-world relationships.^19,21^

This study has limitations. First, it is observational in nature, so causality cannot be inferred. Second, while previous work in similar populations has supported the validity of this approach, it requires self-reporting of sensitive information and we cannot rule out response biases.^22^ Third, the study sample included anyone who reported use of stimulants within the past 6 months, thus only a subset of this population is likely to meet diagnostic criteria for a stimulant use disorder and thus be eligible for such an intervention. This may be one contributor to why rates of prescribing were so low in the overall sample. Also, due to the limited statistical power, we were unable to undertake more advanced analyses of discontinuation of psychostimulant prescriptions. Fourth, prescriptions received were self-reported by participants as having been for the explicit purposes of safer supply, but misclassification of medications received for other indications (ie, attention-deficit/hyperactivity disorder, narcolepsy) is possible. Fifth, the generalizability of this sample to PWUD in different settings is unknown, as are its implications beyond the setting of the COVID-19 pandemic.

## Conclusion

In a community-recruited cohort of people who use stimulants, the prevalence of psychostimulant prescriptions as a harm reduction intervention during a period of “safer supply prescribing” was low, though individual satisfaction levels among those who received prescriptions was much higher. Given recent guidelines which have highlighted psychostimulants as a potential option not only for harm reduction but also as treatment for stimulant use disorders, this work underscores the importance of better understanding which specific individuals might benefit from this type of intervention. Further research on target populations, medication types, dosing strategies, and empiric measurements of effectiveness (or lack thereof) is urgently needed.
